# A Tau PET tracer PBB3 binds to TMEM106B amyloid fibril in brain

**DOI:** 10.1038/s41421-024-00674-z

**Published:** 2024-05-14

**Authors:** Qinyue Zhao, Yun Fan, Wanbing Zhao, You Ni, Youqi Tao, Jiang Bian, Wencheng Xia, Wenbo Yu, Zhen Fan, Cong Liu, Bo Sun, Weidong Le, Wensheng Li, Jian Wang, Dan Li

**Affiliations:** 1 Bio-X Institutes, Key Laboratory for the Genetics of Developmental and Neuropsychiatric Disorders (Ministry of Education), Shanghai Jiao Tong University, Shanghai, China; 2 Department of Neurology and National Research Center for Aging and Medicine & National Center for Neurological Disorders, State Key Laboratory of Medical Neurobiology, Huashan Hospital, Fudan University, Shanghai, China; 3 Interdisciplinary Research Center on Biology and Chemistry, Shanghai Institute of Organic Chemistry, Chinese Academy of Sciences, Shanghai, China; 4 Department of Neurosurgery, Huashan Hospital, Shanghai Medical College, Fudan University, Shanghai, China; 5 State Key Laboratory of Chemical Biology, Shanghai Institute of Organic Chemistry, Chinese Academy of Sciences, Shanghai, China; 6 School of Life Science and Technology, ShanghaiTech University, Shanghai, China; 7 Shanghai University of Medicine & Health Sciences Affiliated Zhoupu Hospital, Shanghai, China; 8 Center for Clinical and Translational Medicine, Shanghai University of Medicine and Health Sciences, Shanghai, China; 9 Department of Anatomy and Histoembryology, School of Basic Medical Sciences, State Key Laboratory of Medical Neurobiology and MOE Frontiers Center for Brain Science, Institutes of Brain Science, Shanghai, China; 10 Zhangjiang Institute for Advanced Study, Shanghai Jiao Tong University, Shanghai, China; 11 WLA Laboratories, World Laureates Association, Shanghai, China

**Keywords:** Cryoelectron microscopy, Protein aggregation

Dear Editor,

Neurodegenerative diseases (NDs) are defined by pathological amyloid aggregates, such as Tau tangles and amyloid β (Aβ) plaques in Alzheimer’s disease (AD)^1,2^. The emergence of positron emission tomography (PET) imaging, using tracers like ^11^C-PBB3 for Tau and ^11^C-PiB for Aβ, offers significant diagnostic promise^3–5^. The challenge in achieving selective binding with PET tracers is exacerbated by the structural similarities among amyloid fibrils^6,7^. The recently identified transmembrane protein 106B (TMEM106B) fibril, prevalent in both NDs and normal elderly individuals, complicates the landscape^8–11^. This revelation prompts critical question regarding whether existing PET tracers, initially designed for Tau and Aβ, might also recognize TMEM106B fibril.

**Fig. 1:**
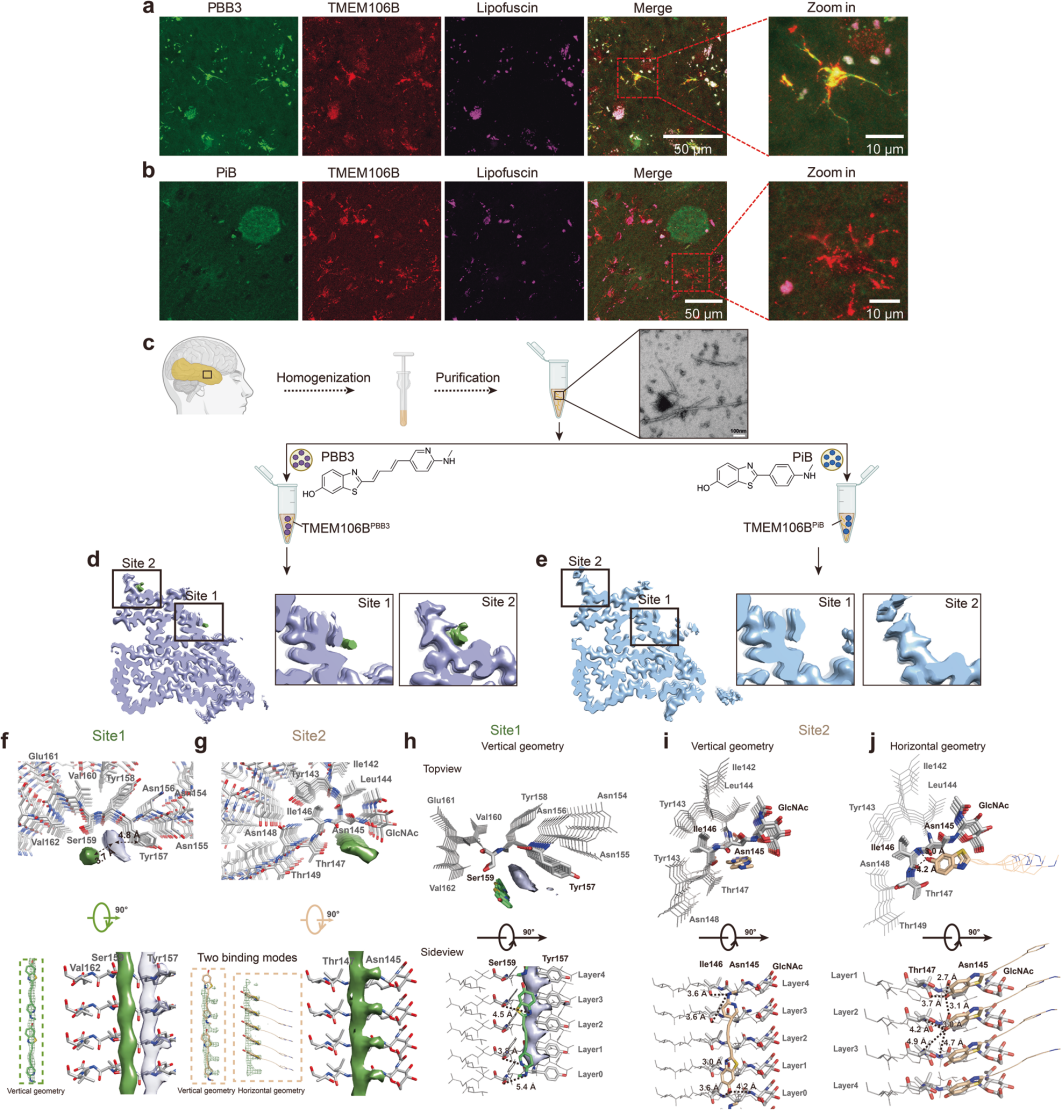
PBB3 binds with TMEM106B fibril. **a** Immunofluorescent staining of TMEM106B (red) and PBB3 (green) in temporal cortex of a centenarian. Zoom-in view illustrates co-localization. **b** Anti-TMEM106B (red) and PiB (green) staining in the same region, with a close-up view of TMEM106B puncta. The intensity of autofluorescent lipofuscin was captured in a separate channel (magenta). **c** Preparation workflow for brain-derived TMEM106B fibrils, confirmed by NS-TEM; includes incubation with PBB3 (TMEM106B^PBB3^) or PiB (TMEM106B^PiB^) for cryo-EM. **d**, **e** Cryo-EM maps of TMEM106B^PBB3^ (light purple) (**d**) and TMEM106B^PiB^ (blue) (**e**) fibrils. **f**, **g** The two extra densities in the TMEM106B^PBB3^ fibril are highlighted in green and magnified. Top and side view of extra densities at site 1 (**f**) and 2 (**g**). Two alternative binding modes of PBB3 are modeled in sticks, atoms without observed density are shown in lines. **h**–**j** Two side views of the interactions between PBB3 and TMEM106B at two sites, detailing binding geometries, interaction residues, and hydrogen bonds, with models and densities colored appropriately.

Here, we first characterized the morphologies of TMEM106B puncta in brain through immunohistochemistry and immunofluorescence. Numerous TMEM106B puncta were presented in the 101-year-old individual’s brain but sparse in the younger control subject (Supplementary Fig. S1). Similar to previous studies^9,12^, two major morphologies were uncovered: cytoplasmic inclusions and short filamentous processes (Supplementary Fig. S2a). Further thioflavin-S also co-stained with TMEM106B puncta, which further validate the aggregated form of TMEM106B (Supplementary Fig. S2b). Except for the TMEM106B puncta, we also confirmed the brain sample of this centenarian contained abundant Aβ plagues, trace amounts of Tau tangles, with no co-localization observed with TMEM106B puncta (Supplementary Fig. S2c, d). Next, using the same brain sample, we investigated if PET tracers, including PBB3 targeting Tau tangles and PiB staining Aβ plagues, could stain TMEM106B puncta. As expected, both tracers could identify their primary targets in this centenarian’s brain slices (Supplementary Fig. S3a, b). Strikingly, we found that PBB3 but not PiB co-stained with TMEM106B puncta, with potential interference from lipofuscin eliminated (Fig. 1a, b; Supplementary Fig. S3c). More strikingly, PBB3-positively signals characterized by short filamentous morphology co-localized well with GFAP-positive staining, which presented with abundant TMEM106B fibrillar aggregates (Supplementary Fig. S4a, b). The observation suggests that the cells doubly stained with PBB3 and TMEM106B are astrocytes.

The co-localization of PBB3 with TMEM106B puncta in the brain led us to delve into their atomic-level interactions using cryo-electron microscopy (cryo-EM). We successfully extracted high-quality TMEM106B fibrils from the same brain region used in immune-staining assays. After incubating the ex vivo TMEM106B fibrils with PBB3 (termed as TMEM106B^PBB3^) and PiB (termed as TMEM106B^PiB^) separately, two samples were applied for cryo-EM structural analysis (Fig. 1c). Two-dimensional (2D) classifications and three-dimensional (3D) density maps indicated that both TMEM106B^PiB^ and TMEM106B^PBB3^ fibril shared high structural similarities to TMEM106B alone (TMEM106B^Apo^) fibril (extracted from brain of the same centenarian) as we previously reported (PDB ID: 7X83) (Fig. 1d, e; Supplementary Figs. S5a–d, S6a and Supplementary Table S1)^8^. Surprisingly, 3D density map of TMEM106B^PBB3^ fibril revealed two additional densities, named site 1 and site 2. Those two additional densities were also absent in other TMEM106B fibrils reported by different labs and extracted from various NDs or aged individuals (Fig. 1d; Supplementary Fig. S6)^8–10^. In contrast, 3D density map of TMEM106B^PiB^ fibril closely mirrored that of TMEM106B^Apo^ fibril (Fig. 1e; Supplementary Fig. S6a). To distinguish the observed extra densities from lower resolution or solvent artifacts, comprehensive density map refinements were undertaken as previously reported^13^. In order to meticulously quantify the extra densities at both binding sites, we divided the final reconstructed particles into 3 (TMEM106B^PBB3^) or 2 (TMEM106B^PiB^) parts, then performed the refinements respectively. It revealed that the extra densities at both binding sites were notably weaker than the density of surrounding protein and amounted approximately 40% relative to the surroundings. When juxtaposed with TMEM106B^PiB^ control group, the relative density ratios were discerned to be around 10% for site 1 and 20% for site 2 (Supplementary Fig. S7 and Table S2). We also compared the density maps of TMEM106B^PBB3^ and TMEM106B^PiB^ complexes at different contour levels, the result further indicated that PBB3, but not PiB, binds to the TMEM106B fibril core, consistent with the results of immuno-staining in brain slices (Fig. 1a, b; Supplementary Fig. S8).

We next build the structural models to investigate the interaction between TMEM106B fibril and PBB3 (Supplementary Figs. S5e, S9 and Table S1). The cryo-EM structure of TMEM106B^PBB3^ revealed two intriguing extra densities situated at grooves of fibril surface, including site 1 pocket, created by Tyr157, Ser159, and Val162, and site 2 pocket, shaped by Asn145 and Thr147 (Fig. 1f, g). Notably, both densities exhibit continuous tubular-like structure along the fibril axis, with the PBB3 molecule fitting into the densities, paralleling to fibril axis and spanning four rungs of TMEM106B. Intriguingly, the PBB3 densities are morphologically similar to those of APN-1607 (a propanol derivative of PBB3) in complex with ex vivo Tau fibrils^13^. This similarity implies that scaffold shared between PBB3 and APN-1607 may selectively engage within grooves of fibrils in a vertical orientation.

At site 1, PBB3 form hydrogen bonds with hydroxyl oxygen of Ser159 through multiple polar atoms. Adjacent to the tubular PBB3 density at site 1, another unidentified tubular density aligns along the fibril axis, with a distance of 3.7 Å from PBB3 and 4.8 Å from Tyr157 of TMEM106B, respectively. Notably, the unidentified density was also observed in the density maps of TMEM106B^Apo^ and TMEM106B^PiB^ fibrils, suggesting it could be an unidentified endogenous ligand that stabilizes the binding of PBB3 to TMEM106B fibrils (Fig. 1h). At site 2, PBB3 interact with hydroxyl oxygen of Asn145 of TMEM106B as well as the amide nitrogen of Asn145, Ile146 and Thr147 via its phenolic hydroxyl group (Fig. 1i). Intriguingly, in addition to the tubular density, an extra density growing from each 4.8 Å rung of the tube was observed. This T-shaped density led the construction of an alternative orientation of PBB3, aligned perpendicularly to the fibril axis. Accordingly, we built the possible binding model. Notably, only the benzothiazole ring of PBB3 fits this density, hinting the highly flexibility in the rest part of PBB3. This model proposes neighboring PBB3 molecules forming T-shaped π–π stacking through their benzothiazole rings, which stabilize PBB3 along the pocket, geometry echoed in the APN-1607-Tau fibril structure (Fig. 1j). Concurrently, N-acetyl-D-glucosamine modification on Asn145 may further stabilize PBB3 (Fig. 1i, j).

PET tracers have been extensively explored to target specific fibrillar aggregates. The recently identified TMEM106B fibrils widely distributed in both ND patients and aging individuals have provoked two pivotal questions: (1) Whether tracers designed for targeting other fibrils may also recognize TMEM106B fibrils? (2) Can a TMEM106B-specifc tracer be developed to reveal its appearance, distribution in human brains? Our findings reveal that Tau PET tracer PBB3 binds to TMEM106B fibrils, suggesting its potential to visualize TMEM106B in AD brains alongside Tau. This observation is crucial given TMEM106B’s prevalence in various NDs. Further investigations outlined the two PBB3 binding sites, located on the conservative handle part of TMEM106B fibril shared among all three ex vivo structural polymorphs, indicating a broad binding capability (Supplementary Fig. S10). Intriguingly, akin to the binding model of APN-1607 with Tau fibrils, PBB3 mainly adopts a vertical binding pattern with limited direct interaction with TMEM106B fibril, which hints at a common interaction mechanism among similar molecules with amyloid fibrils. Differently, although PiB is similar to PBB3 that both contained a benzothiazole substructure, the planner structure of PiB favored a 1:1 molecular stoichiometry parallel orientation to fibril rungs, like previously binding mode between PiB-α-syn complex^14^. Such horizontal binding necessitates complete occupancy in order to yield a clear density, which should cross a higher energy barrier and represented a higher binding affinity. Furthermore, the potential off-target binding of PBB3 to TMEM106B indicates the low binding selectivity of PBB3 to different fibrils^14^. The off-target binding of PBB3 suggests low selectivity among fibrils, but also underscores its potential as a scaffold for creating TMEM106B-specific tracers. Modifying PBB3 to improve selectivity could lead to better understanding of TMEM106B’s role in NDs.

### Supplementary information

Supplementary Information
