# Supplementary material for: A Tau PET tracer PBB3 binds to TMEM106B amyloid fibril in brain
Source: Cell Discov. 2024 May 14;10:50. doi: 10.1038/s41421-024-00674-z (PMC11094151; doi:10.1038/s41421-024-00674-z)
Supplement: Supplementary file 1 — Supplementary Information [file 41421_2024_674_MOESM1_ESM.pdf]

Supplementary information

A Tau PET tracer PBB3 binds to TMEM106B amyloid fibril in brain.

Qinyue Zhao[1¶], Yun Fan[2¶], Wanbing Zhao[2], You Ni[2], Youqi Tao[1], Jiang Bian[1], Wencheng Xia[3], Wenbo Yu[2], Zhen Fan[4], Cong Liu[3], Bo Sun[5], Weidong Le[6,7], Wensheng Li[8], Jian Wang[2]*, Dan Li[1,9,10]*

[1]Bio-X Institutes, Key Laboratory for the Genetics of Developmental and Neuropsychiatric Disorders (Ministry of Education), Shanghai Jiao Tong University, Shanghai, China.

[2]Department of Neurology and National Research Center for Aging and Medicine & National Center for Neurological Disorders, State Key Laboratory of Medical Neurobiology, Huashan Hospital, Fudan University, Shanghai, 200040, China.

[3]Interdisciplinary Research Center on Biology and Chemistry, Shanghai Institute of Organic Chemistry, Chinese Academy of Sciences, Shanghai, 201210, China.

[4]Department of Neurosurgery, Huashan Hospital, Shanghai Medical College, Fudan University, Shanghai, 200040, China.

[5]School of Life Science and Technology, ShanghaiTech University, Shanghai 201210, China

[6]Shanghai University of Medicine & Health Sciences Affiliated Zhoupu Hospital, Shanghai 201318, China

[7]Center for Clinical and Translational Medicine, Shanghai University of Medicine and Health Sciences, Shanghai 201318, China

[8]Department of Anatomy and Histoembryology, School of Basic Medical Sciences, State Key Laboratory of Medical Neurobiology and MOE Frontiers Center for Brain Science, Institutes of Brain Science, Fudan University, Shanghai, 200032, China.

[9]Zhangjiang Institute for Advanced Study, Shanghai Jiao Tong University, Shanghai 200240, China.

[10]WLA Laboratories, World Laureates Association, Shanghai 201203, China

¶These authors contributed equally to this work.

*To whom correspondence should be addressed. E-mails: lidan2017@sjtu.edu.cn and wangjian_hs@fudan.edu.cn.

Materials and Methods

Human brain samples

The brain samples of the 'normal' individual aged 52 years old and undergoing brain trauma surgery were acquired from Brain Disease Biobank of Huashan Hospital. This individuals' family member gave his or her informed consent for the collection of the abandoned brain tissue produced in the surgery. The protocol used was approved by Ethics Committee of Huashan Hospital Affiliated to Fudan University（the project number KY2020-065). The post-mortem brain samples of the centenarian were acquired from the Body Donation Station in Fudan University (Shanghai Red Cross Society). Informed consent was obtained from this donor and his next of kin. Ethical approval of this study was granted by the Human Studies Institutional Review Board, Huashan Hospital, Fudan University. All procedures carried out in this study were in conformity with the ethical standards of Declaration of Helsinki.

Immunohistochemistry of TMEM106B

Immunohistochemistry was performed as we described previously[1]. In brief, brain tissues post-fixed in 4% paraformaldehyde solution (PFA) derived from a 52-year-old normal individual and the centenarian were dehydrated in 20% and 30% sucrose solution sequentially. Then the brain tissues were cut into 20 μm thick sections with cryotome and the brain sections were pre-treated with formic acid, followed by the treatment of hydrogen peroxide for 30 min to quench endogenous peroxidase. Next, the sections were incubated in blocking solution (5% bovine serum albumin, 3% gout serum plus 0.3% Triton X-100 in PBS) for 30 min. Primary antibody of TMEM106B (NBP1-91311, Novus) was incubated overnight at 4 °C. After that, the sections were incubated with HRP polymer anti-rabbit detection antibody (Vector Laboratories). VECTASTAIN ABC Kits (HRP) and VECTASTAIN DAB Substrate Kits (Vector Laboratories) were used to visualize the antigen. Finally, sections were counterstained with hematoxylin.

Immunofluorescence staining and PBB3 labelling

Immunofluorescence staining and PBB3 labelling was performed according to the protocol previously reported[2]. Briefly, post-mortem brain tissue derived from the centenarian was fixed in 4% PFA, followed by gradient dehydration in 20% and 30% sucrose solution, sequentially. Next, the brain tissue was embedded in O.C.T compound (4583, SAKURA) and cut into 20 μm thick sections with cryotome. Brain sections were washed three times with PBS for 5 min at room temperature to remove O.C.T. After blocking and permeabilization using PBS containing 5% BSA and 0.3% Triton X-100, sections were incubated with primary antibody against TMEM106B (NBP1-91311, Novus), Aβ (SIG-39320, Biolegend), p-Tau (MN1020, Invitrogen), GFAP (G3893, Sigma-Aldrich) overnight at 4°C.Then the sections were incubated with secondary fluorescence antibodies at room temperature for 2 h and washed three times with PBS sequentially. After final wash, brain sections used for fluorescence labelling with PBB3 were incubated in 50% ethanol containing 30 μM of PBB3 at room temperature for 30 min. The samples were rinsed with 50% ethanol for 5 min, dipped into distilled water twice for 3 min, and mounted in non-fluorescent mounting media (0100-01; SouthernBiotech). Brain sections unlabeled with PBB3 were directly mounted with non-fluorescent mounting media. Sections were scanned with SP8 confocal microscope. Secondary antibodies used for immunofluorescence were Alexa fluorophore (405, 488, or 568)-coupled goat anti-mouse IgG, Alexa fluorophore (488, 647)-coupled goat anti-rabbit IgG (All from Invitrogen, ThermoFisher Scientific; 1:1000).

ThS labeling of human brain sections

For staining of ThS, brain sections were incubated in 50% ethanol containing 0.05% ThS at RT for 8 min. The samples were rinsed with 50% ethanol for 5 min, dipped into distilled water twice for 3 min, and mounted in ProLong Glass Antifade Mountant (Invitrogen, cat. P36984). Fluorescence images for ThS were captured using SP8 confocal microscope. After that, all sections labelled with ThS were washed in PBS to remove the mounting media and pre-treated with FA, followed by immunostaining of TMEM106B (NOVUS, cat. NBP1-91311; 1:100). Secondary

antibody Alexa Fluor 568 (ThermoFisher Scientific; 1:500) was used for TMEM106B labeling. Images were captured using SP8 confocal microscope.

Chemical synthesis of PBB3 and PiB chemical compounds

PBB3 and PiB were synthesized as the literature[3,4]. Two compounds were dissolved in anhydrous dimethyl sulfoxide (DMSO) to the final concentration at 10 mM as stock solution in -20°C.

Extraction of sarkosyl insoluble TMEM106B fibrils

The extraction of TMEM106B fibrils from the temporal cortex of the normal elder was conducted as we previously reported[1]. Briefly, brain tissues were manually sufficiently homogenized for three times in buffer containing 10 mM Tris-HCl (pH 7.5), 0.8 M NaCl, 10% sucrose, 1 mM EGTA, 0.1% sarkosyl, PMSF and cocktail. Next, homogenates were brought to 2% sarkosyl and incubated at 37°C for 1 h, followed by centrifugation under 10,000 g at 4°C for 10 min. The supernatants (S1) after that were subjected to ultracentrifugation at 100,000 g for 1 h at 4°C. The pellet (P2) acquired from the ultracentrifugation was resuspend with the extraction buffer (1,000 μl/g), followed by a low-speed centrifugation at 3,000g for 5 min at 4°C. Then the supernatants (S3) were diluted to three-fold in buffer consisting of 50 mM Tris-HCl (pH 7.5), 0.15 M NaCl, 10% sucrose and 0.2% sarkosyl. After that, the diluted S3 was entered into ultracentrifugation at 166,000 g for 1 h at 4°C. Then the pellet (P4) containing sarkosyl insoluble TMEM106B fibrils was resuspended with buffer containing 20 mM Tris-HCl, pH 7.4, 50 mM NaCl (100 μl/g). Finally, the P4 sample was incubated with pronase at 25°C for 2 h to digest the amorphous aggregates and remove the fuzzy coat surrounding the TMEM106B fibril before negative staining transmission electron microscopy (NS-TEM), and cryo-EM assay.

Preparation of tracer-bound brain-extracted TMEM106B fibrils

To prepare brain-derived TMEM106B fibril in complex with two tracers (PBB3 and PiB), P4 sample were firstly digested by 0.4 mg/mL pronase at 25ºC for 1 h. Then P4

component were incubated with 150 μM PBB3 or 150 μM PiB chemical compounds for 1 more hour. The concentrations of two tracers were 15 nmol/g tissue. For these two compounds, the final DMSO concentration was 1.5%. The incubation experiments were all performed in the dark.

Cryo-EM sample preparation and data collection

The aqueous solutions of tracers-bound TMEM106B fibrils were applied to glow-discharged holey copper Quantifoil grids (300 mesh, R1.2/1.3). Then, the cryo-EM grids were plunge-frozen in liquid ethane after blotting with filter paper using Vitrobot Mark IV (FEI, Thermo). Cryo-EM micrographs (40 frames per micrograph) were collected on Thermo Fisher Titan Krios G4 cryo transmission electron microscope, operated at 300 kV with a BioContinuu K3 direct detector (Gatan), using a GIF Quantum energy filter (Gatan) with a slit width of 20 eV to remove inelastically scattered electrons. Super-resolution movies were recorded at ×105,000 magnification with a pixel size 0.83 Å $pixel^{-1}$ and the total dose was ~55 e^{-} ^{Å2} with exposure time of 2 s. Automated cryo-EM data collection was performed by using EPU software (Thermo) with defocus values from -1.0 to -2.0 μm.

Image pre-processing and helical reconstruction

40 movie frames per micrograph were corrected for beam induced motion, aligned, dose-weighted, and further binned with a physical pixel size of 0.83 Å using MotionCorr2 1.2.1[5]. CTFFIND-4.1.8 was used to estimate the contrast transfer function of every micrograph[6]. Fibrils were manually picked using the "Manual picking" program in RELION 4.0[7].

Segments for reference-free 2D classification were individually extracted with a box size of 360 pixels (with a pixel size 0.83 Å $pixel^{-1}$) and an inter-box distance of 29.9 Å. Then re-extract the particle with 864 box size and downscaled to 320 pixels. Segments were separated by reference-free 2D classification steps with a decreasing in-plane angular sampling rate (8°, 2°, and 0.5°) and a T=2 regularization parameter. Segments contributing suboptimal 2D class averages were discarded. The apparent

half pitches and initial helical twist angles were calculated through the splicing of 2D class averages. Purified segments that comprise entire helical crossover were used to construct 3D initial de novo models by the relion_helix_inimodel2d program[7].

The generated initial 3D model and segments were applied to perform 3D classification (k=3) with the helical twist and rise parameters from the splicing of 2D class averages. Local search of symmetry to optimize the helical twist and rise was carried out after separation of β-strands was shown to select the clearest class. Then we performed Bayesian polishing and contrast transfer function refinement to further improve the resolution of 3D reconstruction maps. The final reconstruction map was sharpened in using "Post-processing" program with a soft-edge solvent mask[7]. Overall resolution estimation was calculated based on the gold-standard 0.143 Fourier shell correlation (FSC) between the two independently refined half-maps. Local resolution was estimated using the "Local resolution" program in RELION 4.0 with the same mask and B-factor in post-processing[7].

Model building and refinement

According to the density map after post-processing, the atomic model of TMEM106B fibrils in two tracer-bound fibril complexes were built based on map and structure model of type 1 fibril as previously reported in COOT (PDB:7X83)[1]. Then the ligand bound TMEM106B models were manually adjusted in WinCoot 0.8.9.2[8], followed by refinement against the corresponding map by phenix.real_space_refine program in PHENIX 1.13 with rotamer, Ramachandran, and geometry restraints[9]. The coordinates and geometry restrains of PBB3 were generated using SMILES string in phenix.elbow[9]. Then the ligand was manually docked into the central region of the sharpened density map using Chimera 1.13.1. Final the models were validated by "Comprehensive validation" procedure in PHENIX 1.13[9]. There are more additional details in Table 1.

Quantification of extra densities

For each group, the initial reconstructions with a box size of 320 pixel and pixel size

of 0.83 Å were up-sampled to 503 pixels. This up-sampling was critical to ensure that an integer number of pixels could be extracted for the central 30% Z length of the filament, in line with previously established protocols[10]. The final sharpened maps were restricted into 18 layers for later analysis conveniently and unified. For control datasets, 3 independent refinements were performed by dividing the up-sampled particles into 3 parts randomly in RELION. For the PBB3 group, 2 independent refinements were carried out in the same way, albeit with fewer particles in the final reconstruction. The final 3D auto-refine maps and parameters served as a reference for all three or two independent refinements, respectively. Each reconstruction was post-processed, with a low-pass filter of 15 Å and a B-factor of -90 Å2. For precise quantification, binary masks were created for the different binding sites in each of the half maps. In the solvent region, these masks were extended by 29 pixels around the entire filament, ensuring comprehensive coverage. The areas corresponding to residues 155-164 (for site 1) and 145-151 (for site 2) were designated as the protein density regions for analysis. The relative densities were calculated using following formula:

$$Relative\ binding\ density = \frac{\text{Max (binding site)} - \text{Avg (solvent)}}{\text{Max (protein)} - \text{Avg (solvent)}}$$

In this formula, 'Max (binding site)' represents the highest pixel value within the mask for the binding site; 'Avg (solvent)' is the average pixel value in the solvent region; and 'Max (protein)' denotes the highest pixel value in the protein density for residues 155-164 (for site 1) or 145-151 (for site 2). Unpaired two-tailed t-test was performed for statistical analysis.

Data availability

The cryo-EM maps have been deposited in the Electron Microscopy Data Bank (EMDB) under accession numbers EMD-36043 for TMEM106B^{PBB3} and EMD-36045 for TMEM106B^{PiB}, respectively. The corresponding refined atomic models of the

TMEM106B^{PBB3} fibril and TMEM106B^{PiB} fibril have been deposited in the Protein Data Bank (PDB) following accession numbers 8J7N and 8J7P, respectively. The density maps used are available in EMDB databased under accession number EMD-33054, EMD-26278 and EMD-14189. The structural models used in this study are available in the PDB database under accession codes 7X84 (TMEM106B^{Apo}, TMEM106B type 1), 7QWM (TMEM106B type 1), 7U15, 7X83 (TMEM106B type 1), 7QVC (TMEM106B type 2), 7U13 (TMEM106B type 2), 7SAQ (TMEM106B type 2), 7TMC (TMEM106B type 2), 7X84 (TMEM106B type 2), 7QWL (TMEM106B type 3), and 7QWG (TMEM106B type 2).

Supplementary Figures

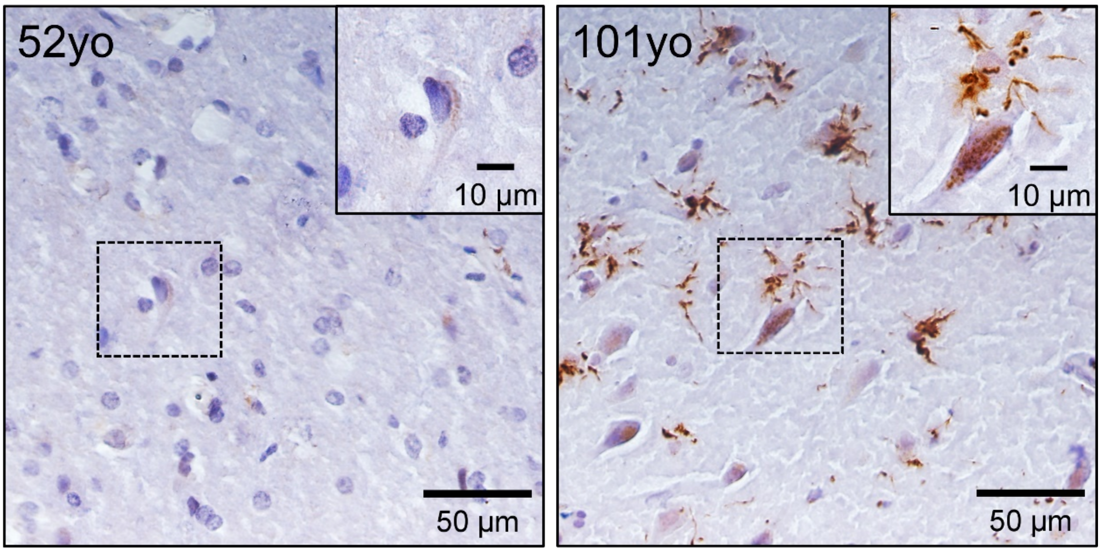

Supplementary Fig. S1. Immunohistochemistry of TMEM106B in brains.

TMEM106B immunostaining of brain slices from the temporal cortex of the centenarian (101-year-old) on the right and a controlled younger individual (52-year-old) on the left. Brain slices were stained with an antibody targeting residue 204-253 of TMEM106B (NBP1-91311, Novus). The cytoplasmic inclusions around nucleus and the short filamentous processes in the centenarian were zoomed in on the right corner. Without noticing similar inclusions in the 52 years old control individual, slight and diffused staining of TMEM106B were observed in (left). yo, years old.

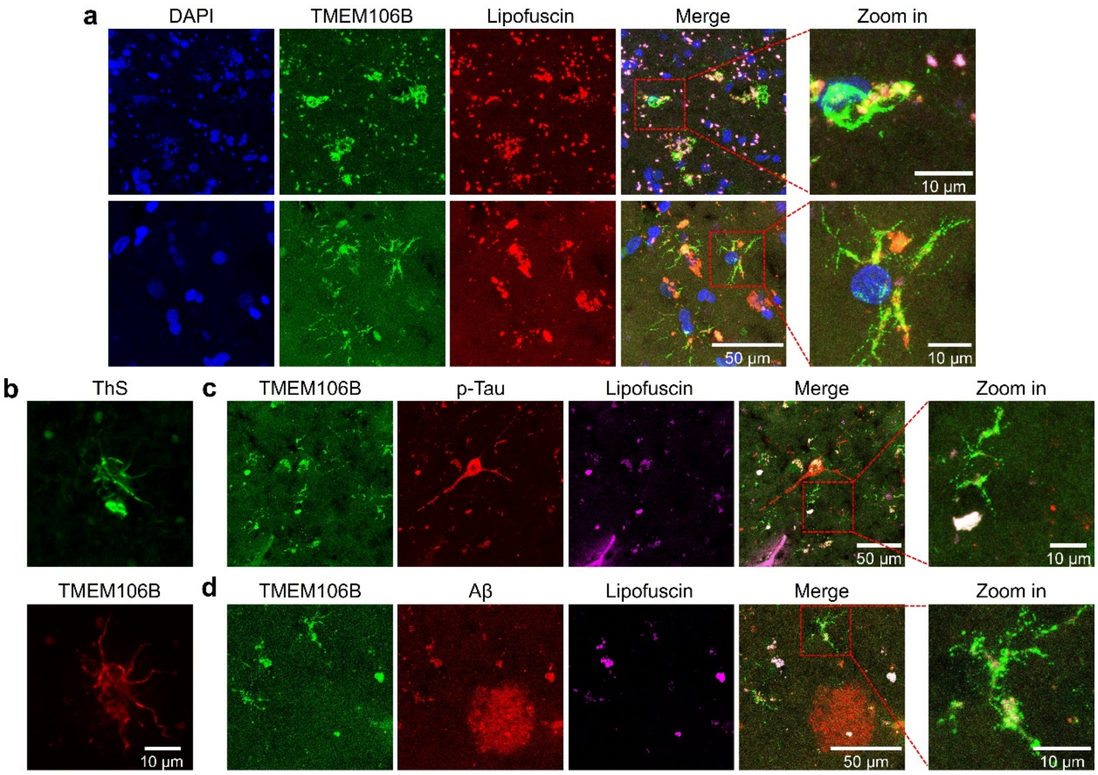

Supplementary Fig. S2. Immunofluorescence staining of TMEM106B, Aβ and p-Tau in the brain sections of the centenarian.

a. Immunofluorescence staining with anti-TMEM106B (residues 204-253) of temporal cortex from the centenarian. Cytoplasmic TMEM106B puncta around nucleus (top) and short filamentous processes (bottom) were zoomed in. **b**. Sequential immunofluorescence staining of ThS and TMEM106B fibrillar aggregates. The brain sections of temporal lobe were firstly incubated with ThS, following by the capture of the representative images of ThS-positive signal. Next, the brain sections were pre-treated with formic acid to enhance epitope exposure as well as quenching ThS-positive intensity and then incubated with anti-TMEM106B primary antibody. Finally, the representative images of TMEM106B fibrillar aggregates were captured by SP8 confocal microscope. Double staining of TMEM106B & p-Tau, and TMEM106B & Aβ in the temporal cortex of the centenarian was shown in **(c)** and **(d),** respectively. Without observing the co-localization between TMEM106B stained short filamentous processes or cytoplasmic puncta and p-Tau and Aβ. TMEM106B inclusions were zoomed in **(c, d)**. Autofluorescent lipofuscin was acquired using a

separate channel which was not used for detecting DAPI, TMEM106B, p-Tau and Aβ.

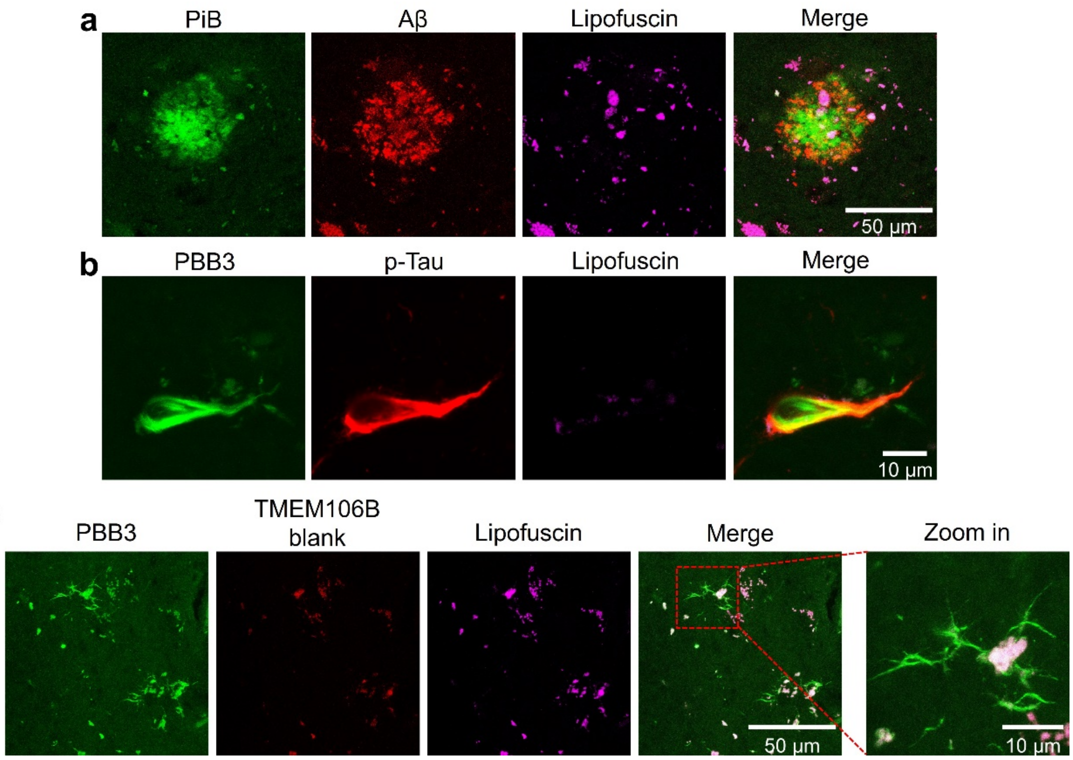

Supplementary Fig. S3. Double staining of the two PET tracers and different amyloid puncta in the centenarian brain sample.

a. Co-staining of PiB and Aβ plaques. **b.** Co-staining of PBB3 and p-Tau. **c.** Immunostaining of control group for PBB3 and TMEM106B double staining which was performed in the absence of anti-TMEM106B antibody. PBB3 positive puncta were zoomed in. The intensity of autofluorescent lipofuscin in (**a-c**) was captured in a separate channel (magenta).

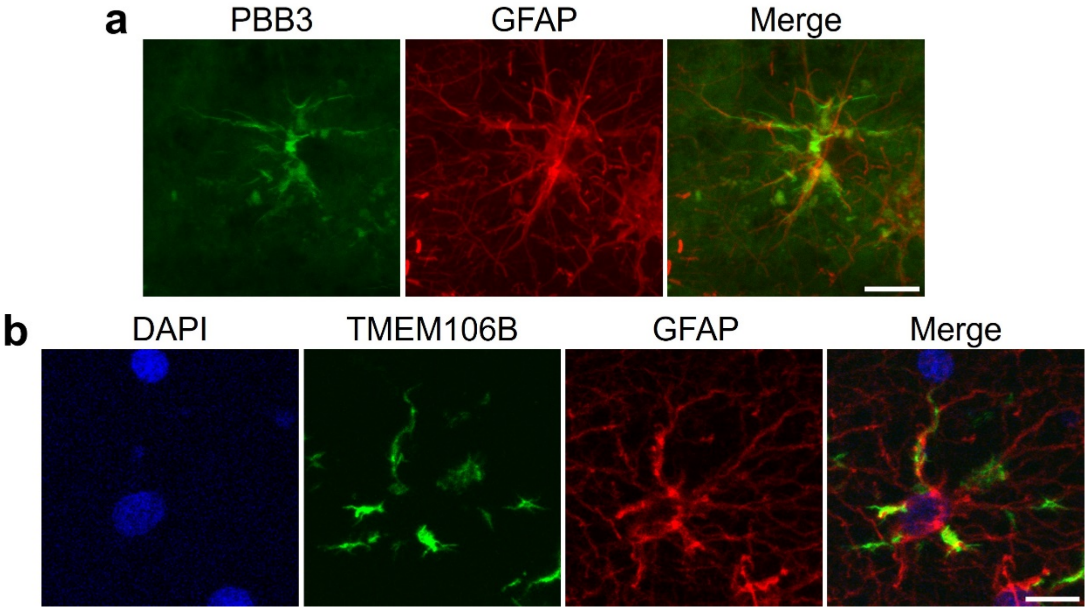

Supplementary Fig. S4. Double immunofluorescence staining of GFAP with PBB3 or TMEM106B.

Double immunofluorescence staining of astrocyte marker-GFAP with PBB3 (**a**) and TMEM106B fibrillar aggregates (**b**), respectively. The brain sections of the temporal cortex from the centenarian were used. Scale bar, 10 μm.

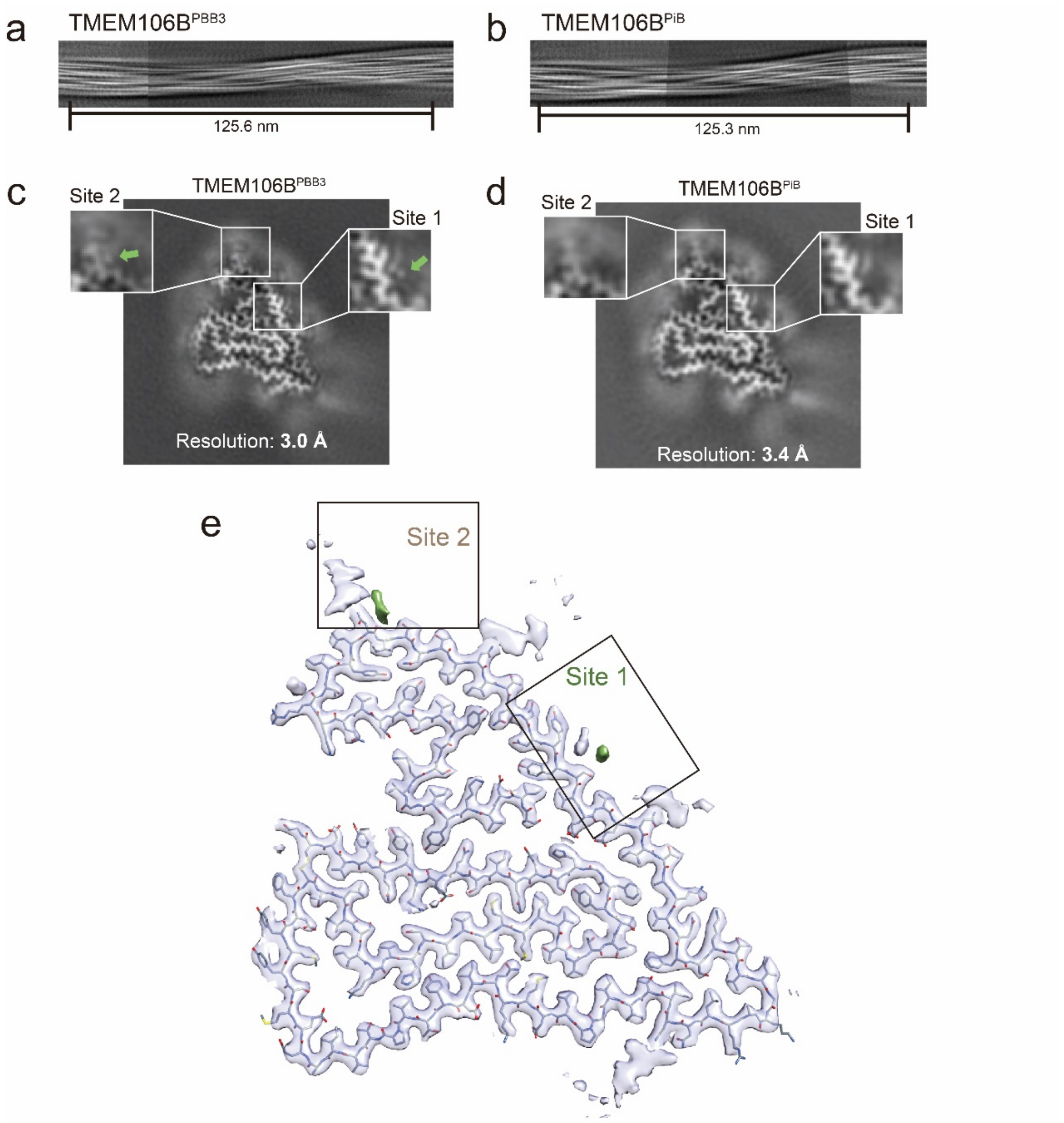

Supplementary Fig. S5. Preparation and cryo-EM structure determination of TMEM106B^{PBB3} and TMEM106B^{PiB} fibrils.

a., **b.** The 2D class averages of TMEM106B^{PBB3} (**a**) and TMEM106B^{PiB} (**b**) fibrils. **c., d.** Central slices of the cryo-EM 3D density maps of TMEM106B^{PBB3} **(c)** and TMEM106B^{PiB} **(d)** fibrils with binding sites of PBB3 zoomed in. The additional densities only existed in TMEM106B^{PBB3} fibril were indicated by green arrow. **e.** Cross-section view of the structural model of TMEM106B^{PBB3} fibril in the density map. The model of the TMEM106B is colored in grey. Two extra densities are colored in green and framed out, named site 1 and site 2.

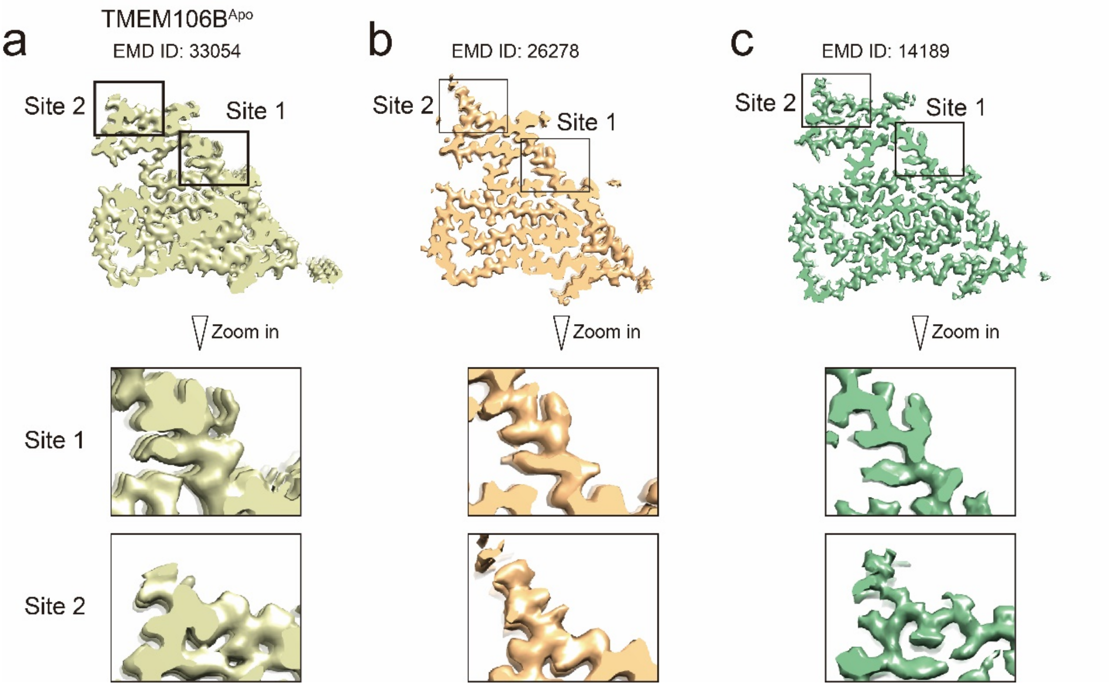

Supplementary Fig. S6. The two extra densities existed in TMEM106B^{PBB3} fibril are absent in other previously identified Type 1 TMEM106B fibril.

Cross-section density maps of three *ex vivo* type 1 TMEM106B fibrils extracted from the same centenarian **(a)**, MSA patient **(b)** and FTLD patient **(c)**. The density map of TMEM106B^{Apo} (EMD ID: EMD-33054) is colored in Kelly, and the other two reported by Chang et al. and Schweighauser et al. are depicted in orange and forest green, respectively. The identical magnified view of site 1 and site 2 were also zoomed in for these three density maps.

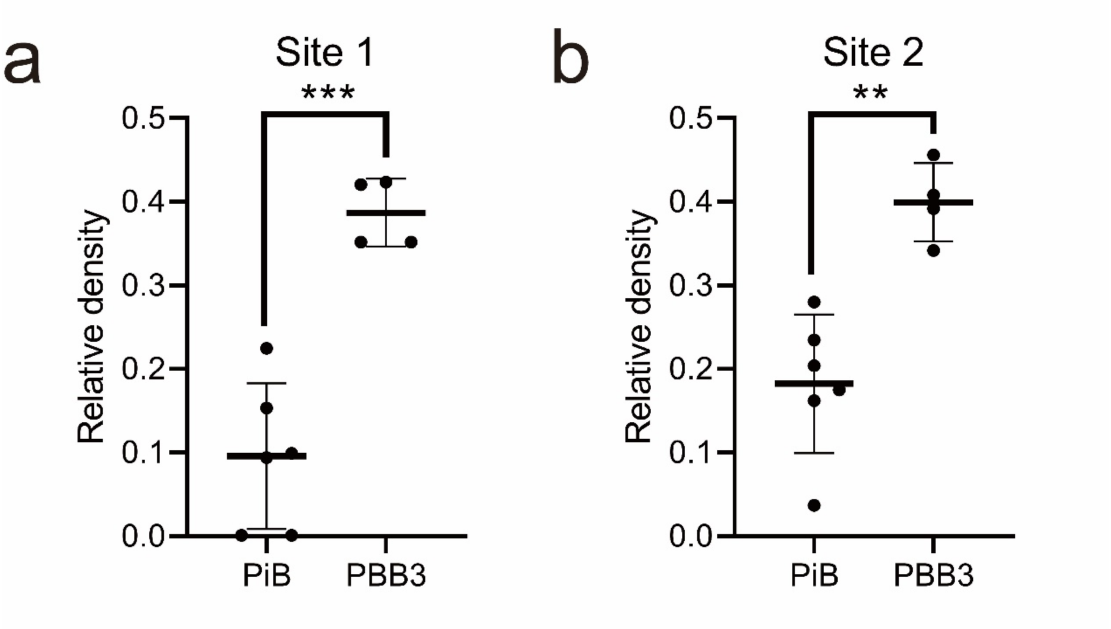

Supplementary Fig. S7. Relative densities at binding sites 1, 2 in cryo-EM maps of TMEM106B^{PBB3} and TMEM106B^{PiB}.

The statistical analysis of relative density at two sites in the density maps of TMEM106B^{PBB3} and TMEM106B^{PiB} (control) fibrils. Means, standard deviations and individual values of 6 half-set reconstructions (for TMEM106B^{PiB}) and 4 half-set reconstructions (for TMEM106B^{PBB3}) are shown. Unpaired two-tailed t-test: *** ($p=0.0003$) for site 1 and ** ($p=0.0015$ for site 2).

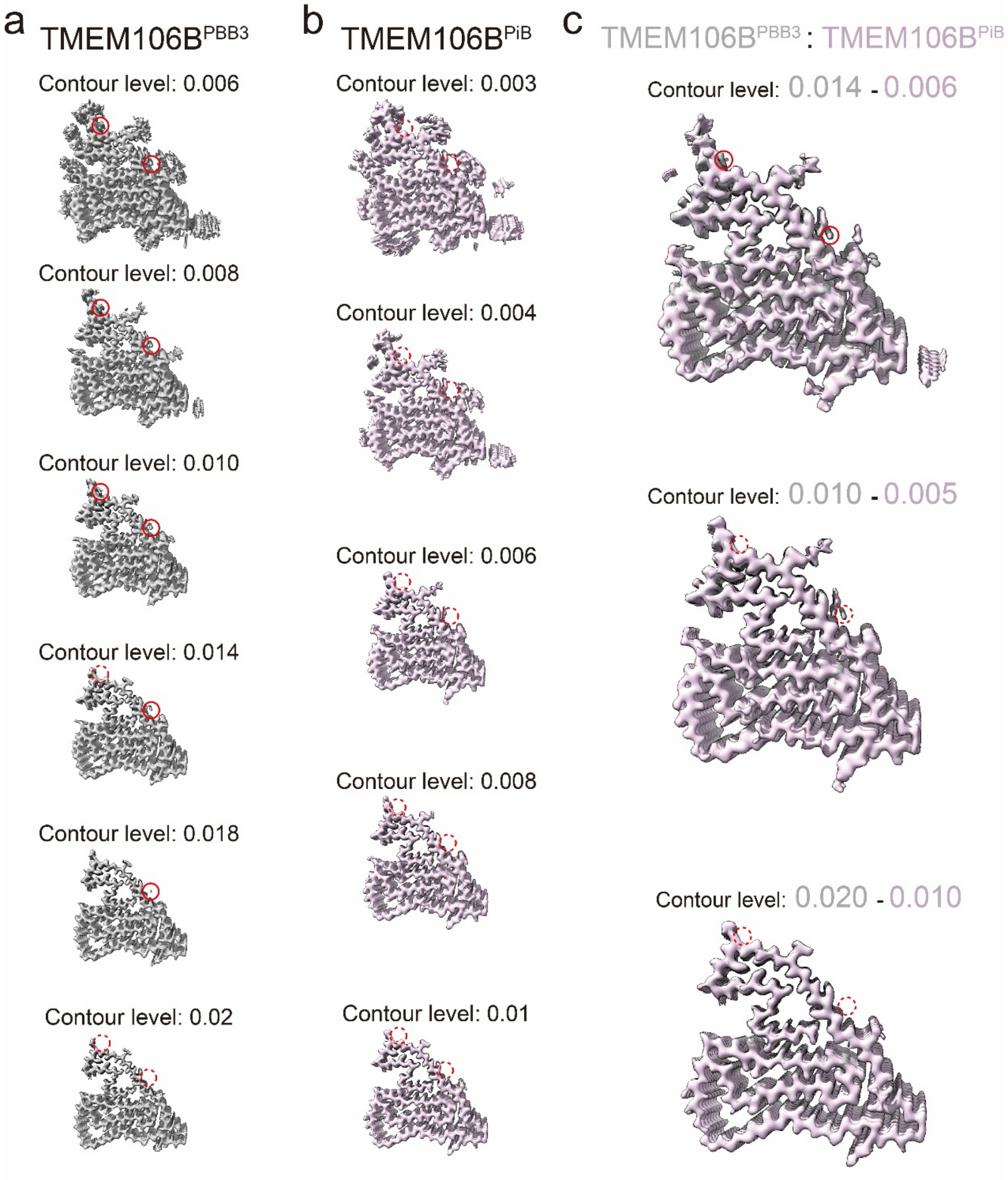

Supplementary Fig. S8. Comparative density maps of TMEM106B^{PBB3} and TMEM106B^{PiB} complexes at varied contour levels.

a. The cryo-EM maps of TMEM106B^{PBB3} fibril at a range of contour levels (0.006-0.02) are presented, with the extra densities at site 1 and site 2 and the disappeared density labeled with solid and dash red circles, respectively. **b**. Cryo-EM maps of TMEM106B^{PiB} fibril are illustrated at different contour levels (0.003-0.01). **c.** Overlay of two maps at varied contour level. The thresholds of two maps are adjusted to align the main chain at different contour levels. On the top (soft contour level), two extra densities on the

TMEM106B^{PBB3} fibril are obviously visible and circled. In the middle panel (moderate contour level), the density at site 2 disappeared, while site 1 still existed. The bottom panel (harsh contour level) presents two extra densities that are invisible in both maps. The density map of TMEM106B^{PBB3} is depicted in grey, and that of TMEM106B^{PiB} is colored in pink.

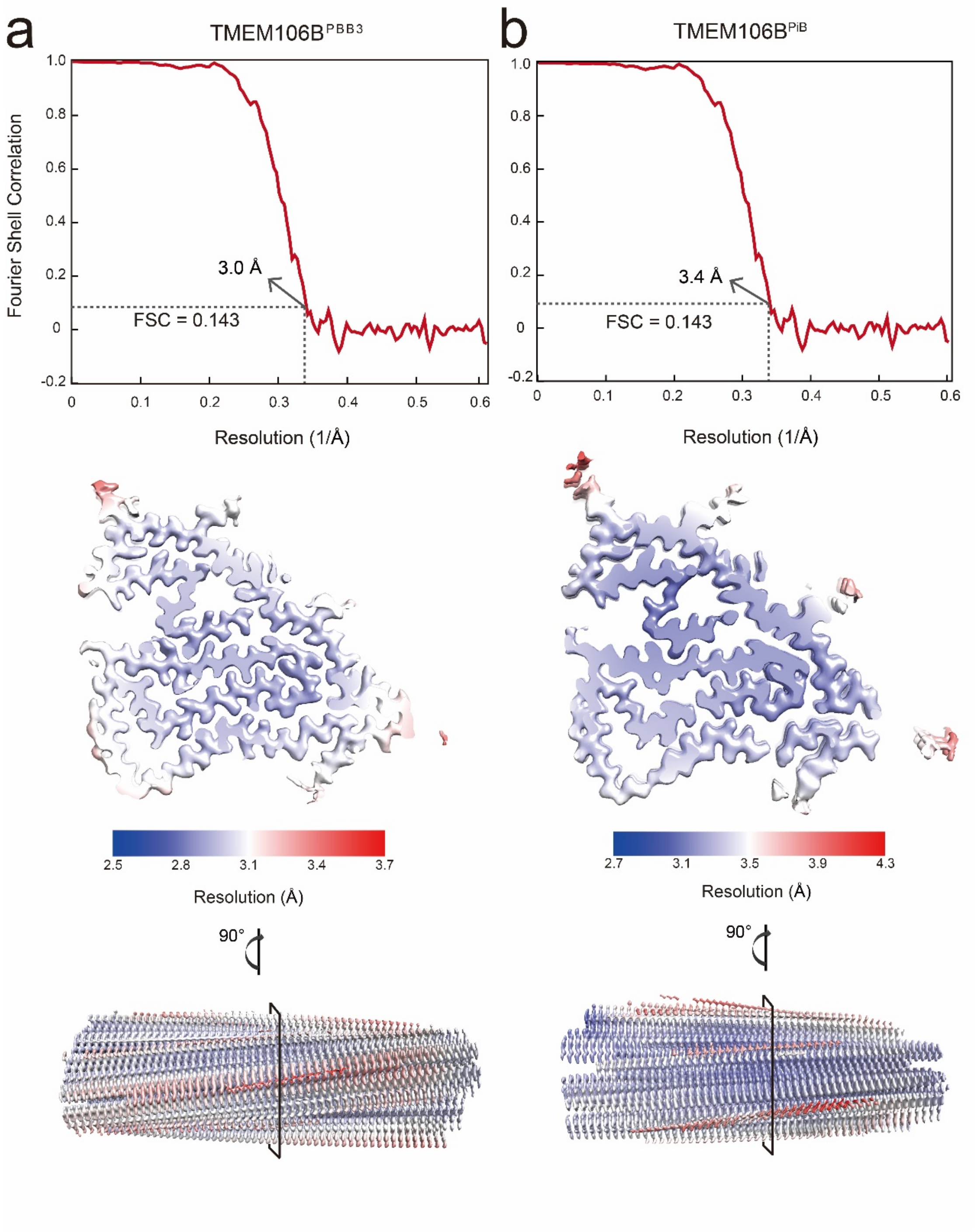

Supplementary Fig. S9. Local resolution estimations of the cryo-EM density maps of TMEM106B^{PBB3} and TMEM106B^{PiB} fibrils.

The overall resolution estimates calculated based on the gold-standard 0.413 FSC of two independently refined half-maps (top) and local resolution plots of the density maps estimated by the "Local resolution" program in RELION (bottom) of TMEM106B^{PBB3} (**a**) and TMEM106B^{PiB} (**b**).

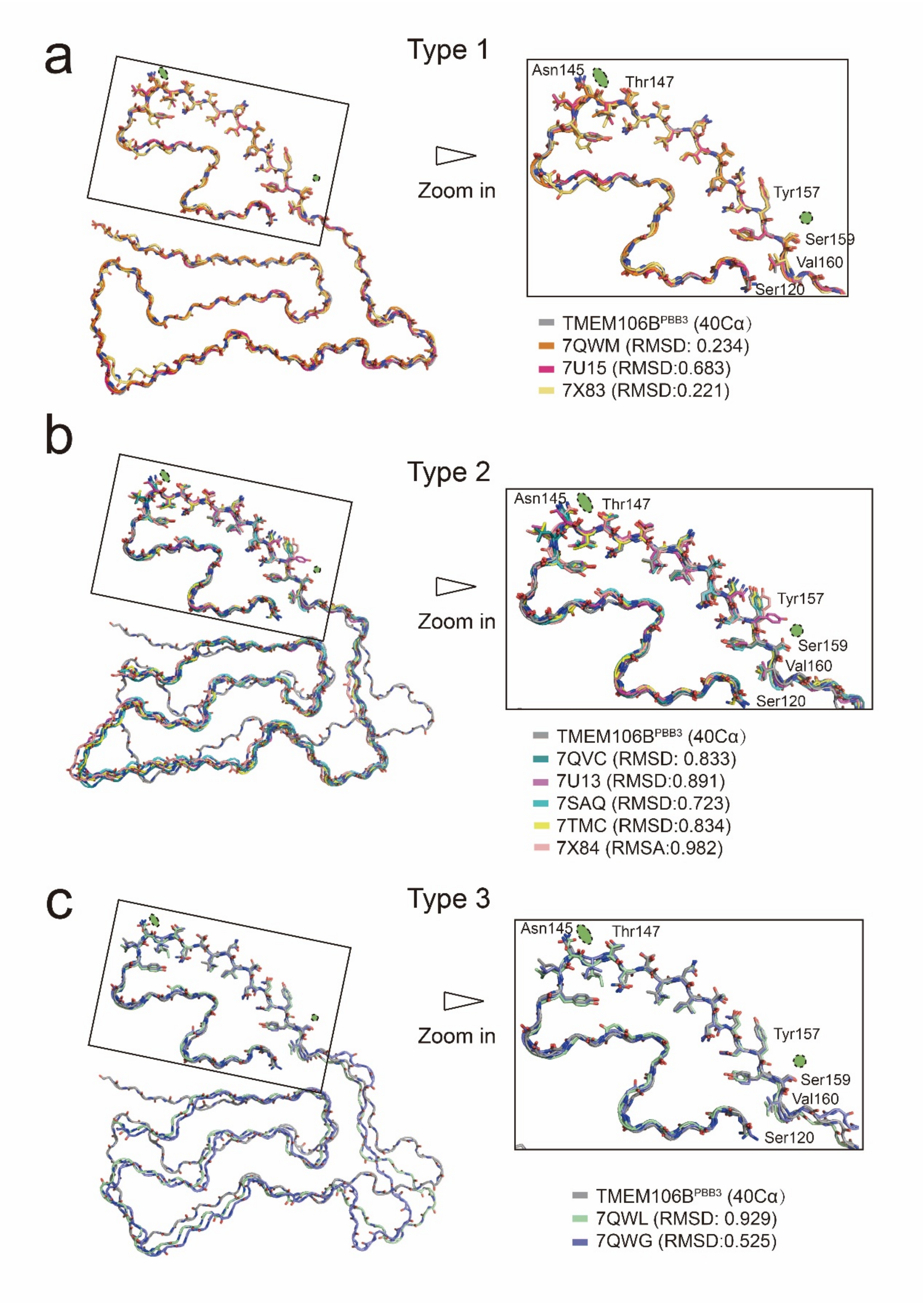

Supplementary Fig. S10. Structural comparison of different TMEM106B polymorphs.

Structural comparison of all the three TMEM106B fibril polymorphs, including Type 1 **(a)**, Type 2 **(b)** and Type 3 **(c)**[1,11-13]. Overlay different types of TMEM106B fibril

from Ser120 to Val 160 at N terminal, the structures are nearly identical with an RMSD less than 1 Å, demonstrating the structural conservation in the N terminal of TMEM106B fibrils. The part using for comparison is zoomed in on the right panel with the possible PBB3 binding site identified above showed in dotted green circles. The surrounding key residues are depicted.

Supplementary Table

Supplementary Table S1. Cryo-EM structural determination and model statistics

Data collection and processing	**TMEM106B^{PBB3}** (EMD-36043) (PDB 8J7N)	**TMEM106B^{PiB}** (EMD-36045) (PDB 8J7P)
**Data Collection**		
Magnification	105,000	105,000
Pixel size (Å)	0.83	0.83
Defocus Range (μm)	-1.0 to -2.0	-1.0 to -2.0
Voltage (kV)	300	300
Camera	BioContinuum K3	BioContinuum K3
Microscope	Krios G4	Krios G4
Exposure time (s/frame)	0.05	0.05
Number of frames	40	40
Total dose ($e^-/Å^2$)	55	55
**Reconstruction**		
Micrographs	3,216	1,334
Manually picked fibrils	3,028	5,726
Box size (pixel)	320	320
Inter-box distance (Å)	26.6	26.6
Initial particle images (no.)	71,570	128,186
Final particle images (no.)	14,665	84,488
Resolution (Å)	3.0	3.4
Map sharpening B-factor ($Å^2$)	-90.4	-136.9
Helical rise (Å)	-0.69	-0.69
Helical twist (°)	4.84	4.83
**Atomic model**		
Non-hydrogen atoms	3,258	3,258
Protein residues	405	405
Ligands	0	0
r.m.s.d. Bond lengths	0.008	0.009
r.m.s.d. Bond angles	0.816	0.802
All-atom clash score	16.13	15.98
Rotamer outliers	0%	0%
Ramachandran Outliers	0%	0%
Ramachandran Allowed	13.53	15.04
Ramachandran Favored	86.47	84.96

Supplementary Table S2. Statistical analysis of the relative density at site 1 and 2.

Binding site	Ligand	Mean	S.D.	n	Unpaired two-tailed t test			F test
					P	T	Degrees of freedom	P
Site 1	PBB3	0.3870	0.1559	4	0.0003	6.171	8	0.2376
	PiB	0.0955	0.0473	6				
Site 2	PBB3	0.3995	0.1653	4	0.0015	4.703	8	0.3790
	PiB	0.1822	0.1040	6				

References

1 Fan, Y. *et al.* Generic amyloid fibrillation of TMEM106B in patient with Parkinson's disease dementia and normal elders. *Cell Res* **32**, 585-588, doi:10.1038/s41422-022-00665-3 (2022).

2 Ono, M. *et al.* Distinct binding of PET ligands PBB3 and AV-1451 to tau fibril strains in neurodegenerative tauopathies. *Brain* **140**, 764-780, doi:10.1093/brain/aww339 (2017).

3 Wang, M., Gao, M., Xu, Z. & Zheng, Q. H. Synthesis of a PET tau tracer [(11)C]PBB3 for imaging of Alzheimer's disease. *Bioorg Med Chem Lett* **25**, 4587-4592, doi:10.1016/j.bmcl.2015.08.053 (2015).

4 Cui, M. *et al.* Synthesis and evaluation of novel (1)(8)F labeled 2-pyridinylbenzoxazole and 2-pyridinylbenzothiazole derivatives as ligands for positron emission tomography (PET) imaging of beta-amyloid plaques. *J Med Chem* **55**, 9283-9296, doi:10.1021/jm300973k (2012).

5 Zheng, S. Q. *et al.* MotionCor2: anisotropic correction of beam-induced motion for improved cryo-electron microscopy. *Nat Methods* **14**, 331-332, doi:10.1038/nmeth.4193 (2017).

6 Rohou, A. & Grigorieff, N. CTFFIND4: Fast and accurate defocus estimation from electron micrographs. *J Struct Biol* **192**, 216-221, doi:10.1016/j.jsb.2015.08.008 (2015).

7 Zivanov, J. *et al.* A Bayesian approach to single-particle electron cryo-tomography in RELION-4.0. *Elife* **11**, doi:10.7554/eLife.83724 (2022).

8 Emsley, P., Lohkamp, B., Scott, W. G. & Cowtan, K. Features and development of Coot. *Acta Crystallogr D Biol Crystallogr* **66**, 486-501, doi:10.1107/S0907444910007493 (2010).

9 Adams, P. D. *et al.* PHENIX: a comprehensive Python-based system for macromolecular structure solution. *Acta Crystallogr D Biol Crystallogr* **66**, 213-221, doi:10.1107/S0907444909052925 (2010).

10 Shi, Y. *et al.* Cryo-EM structures of tau filaments from Alzheimer's disease with PET ligand APN-1607. *Acta Neuropathol* **141**, 697-708, doi:10.1007/s00401-021-02294-3 (2021).

11 Schweighauser, M. *et al.* Age-dependent formation of TMEM106B amyloid filaments in human brains. *Nature*, doi:10.1038/s41586-022-04650-z (2022).

12 Chang, A. *et al.* Homotypic fibrillization of TMEM106B across diverse neurodegenerative diseases. *Cell* **185**, 1346-1355.e1315, doi:10.1016/j.cell.2022.02.026 (2022).

13 Jiang, Y. X. *et al.* Amyloid fibrils in disease FTLD-TDP are composed of TMEM106B not TDP-43. *Nature*, doi:10.1038/s41586-022-04670-9 (2022).
